# Antimicrobial Effects of Chemically Functionalized and/or Photo-Heated Nanoparticles

**DOI:** 10.3390/ma12071078

**Published:** 2019-04-02

**Authors:** Luigia Pezzi, Alfredo Pane, Ferdinanda Annesi, Maria Adele Losso, Alexa Guglielmelli, Cesare Umeton, Luciano De Sio

**Affiliations:** 1 CNR-Lab. Licryl, Institute NANOTEC, 87036 Arcavacata di Rende, Italy; alfredo.pane@cnr.it (A.P.); ferdinanda.annesi@cnr.it (F.A.); alexa.guglielmelli@unical.it (A.G.); cesare.umeton@fis.unical.it (C.U.); 2 DiBEST, University of Calabria, 87036 Arcavacata di Rende, Italy; maria_adele.losso@unical.it; 3 Department of Physics, University of Calabria, Arcavacata di Rende, 87036 Cosenza, Italy; 4 Department of Medico-surgical Sciences and Biotechnologies, Sapienza University of Rome, Corso della Repubblica 79, 04100 Latina, Italy

**Keywords:** antibacterial agents, antibiotic resistance, gold nanoparticles, plasmonic resonance, thermal inactivation

## Abstract

Antibiotic resistance refers to when microorganisms survive and grow in the presence of specific antibiotics, a phenomenon mainly related to the indiscriminate widespread use and abuse of antibiotics. In this framework, thanks to the design and fabrication of original functional nanomaterials, nanotechnology offers a powerful weapon against several diseases such as cancer and pathogenic illness. Smart nanomaterials, such as metallic nanoparticles and semiconductor nanocrystals, enable the realization of novel drug-free medical therapies for fighting against antibiotic-resistant bacteria. In the light of the latest developments, we highlight the outstanding capabilities of several nanotechnology-inspired approaches to kill antibiotic-resistant bacteria. Chemically functionalized silver and titanium dioxide nanoparticles have been employed for their intrinsic toxicity, which enables them to exhibit an antimicrobial activity while, in a different approach, photo-thermal properties of metallic nanoparticles have been theoretically studied and experimentally tested against several temperature sensitive (mesophilic) bacteria. We also show that it is possible to combine a highly localized targeting with a plasmonic-based heating therapy by properly functionalizing nanoparticle surfaces with covalently linked antibodies. As a perspective, the utilization of properly engineered and chemically functionalized nanomaterials opens a new roads for realizing antibiotic free treatments against pathogens and related diseases.

## 1. Antimicrobial Resistance

Chronic diseases and mortality are consequences of bacterial infections that represent a burden to health and global economics. For their efficiency and effectiveness, antibiotics, exploited as antimicrobial agents, represent a primary treatment method; however, since the first time they were released into the environment, their successful use has been compromised by the development of tolerance and/or resistance. Currently, multidrug resistance (MDR), related to the indiscriminate widespread use and abuse of antibiotics has become, in fact, a serious global health problem, with devastating consequences for patient care [1].

**Figure 1:**
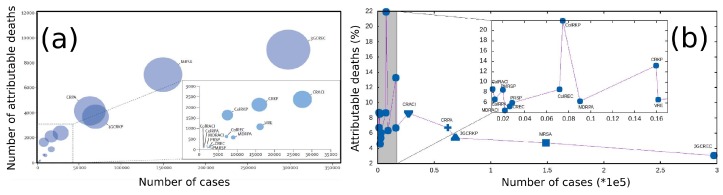
Infections with antibiotic-resistant bacteria, EU and European Economic Area, 2015. (**a**) The diameter of bubbles represents the number of disability-adjusted life-years [2]. (**b**) Each blue point represents antibiotic-resistant bacteria in EU and European Economic Area in 2015. Incidence cases are reported on the *x*-axis, the relative percentage of attributable deaths is reported on the *y*-axis. Bacteria are the same as in (**a**). ColRACI = *colistin-resistant Acinetobacter* spp. CRACI = *carbapenem-resistant Acinetobacter* spp. MDRACI = *multidrug-resistant Acinetobacter* spp. VRE = *vancomycin-resistant Enterococcus faecalis and Enterococcus faecium*. ColREC = *colistin-resistant Escherichia coli*. CREC = *carbapenem-resistant E. coli*. 3GCREC = *third-generation cephalosporin-resistant E. coli*. ColRKP = *colistin-resistant Klebsiella pneumoniae*. CRKP = *carbapenem-resistant K pneumoniae*. 3GCRKP = *third-generation cephalosporin-resistant K pneumoniae*. ColRPA = *colistin-resistant Pseudomonas aeruginosa*. CRPA = *carbapenem-resistant P aeruginosa*. MDRPA = *multidrug-resistant P aeruginosa*. MRSA = *meticillin-resistant Staphylococcus aureus*. PRSP = *penicillin-resistant Streptococcus pneumoniae*. PMRSP = *penicillin-resistant and macrolide-resistant S pneumoniae* Reprinted with permission from [2].

The number of MDR bacterial strains has increased significantly and, although many financial resources have been invested, MDR has become faster than the development of new classes of antibiotic molecules. This problem has not only clinical but also economic implications, as reported in a recent study [2] where authors estimate the health burden caused in EU and European Economic Area (EEA) in 2015 by antibiotic-resistant bacteria. The study measured the impact of the phenomenon in terms of number of cases, number of attributable deaths and disability-adjusted life years (DALYs), calculated as the sum of “Years of Life Lost” and “Years of Life lived with Disability”. Eight bacterial species frequently isolated from blood or cerebrospinal fluid have been included in this study: *Acinetobacter* spp.; *Enterococcus faecalis* and *faecium*; *Escherichia coli*; *Klebsiella pneumoniae*; *Pseudomonas aeruginosa*; *Staphylococcus aureus* meticillin-resistant (MRSA) and *Streptococcus pneumoniae*. Results showed that 67.9% of DALYs were caused by infections with *E. coli* and *K. pneumoniae* (resistant to cephalosporins of third-generation), *S. aureus* (methicillin-resistant) and *P. aeruginosa* (carbapenem-resistant); bacteria resistant to carbapenems or colistin are responsible for 38.7% of DALYs. Authors showed also that every age is involved in infections, significantly in childhood. In adults, the burden increases with age, suggesting that the overall ageing of population in EU and EEA entails a worsening of the problem. Figure 1a shows the number of attributable deaths, in 2015, as a function of the number of cases in EU and European Economic Area for each bacterial species under analysis. Diameter of bubbles represents DALYs. From data reported in Table 1 of that study [2], we inferred Figure 1b, which shows the association between the number of cases in EU and European Economic Area in 2015 and the percentage of deaths for each antibiotic-resistant bacteria. Figure 1a,b show that the effect on health of antibiotic-resistant bacteria is related not only to virulent infections with large incidences, and therefore large DALYs values, (bubbles diameters in Figure 1a), but also to low incidence infections like carbapenem-resistant *K. pneumoniae*, a case where the percentage of attributable mortality may result very high (see Figure 1b).

In order to understand how MDR develops, we have to recall the functioning mechanism of antibiotics, which interact with their molecular targets to modify or block the normal bacteria functions by exploiting different action. One of the principal antibacterial actions is the inhibition or regulation of the enzymes involved in cell wall biosynthesis, as β-lactams antibiotics and glycopeptides do. Antibiotics can also interfere with cell biochemical reactions (protein synthesis, macrolide, metabolites), such as the folic acid, sulfonamides, and the nucleic acid synthesis do. On the other hand, microorganisms show different alternatives to avoid the action of antibiotics, like the production of inactivating antibiotic enzymes, such as β-lactamase, alteration of the permeability of the envelope, alteration of the target site, alternative metabolic pathways, biofilm formation [3]. As a matter of fact, antimicrobial resistance to antibiotics is an ancient phenomenon occurring in the environment as a passage of the evolutionary process [4] and represents not only a natural consequence of antibiotic use, but also an intrinsic capacity of bacteria to survive in a hostile environment, due to their genetic plasticity [5]. Furthermore, in addition to their intrinsic and adaptive [6] resistance to a particular antimicrobial agent as a consequence of structural or functional characteristics, and to silent mutations, bacteria have evolved sophisticated mechanisms to acquire, transfer and spread resistance genes. The horizontal transfer of genes (HGT) is mediated, classically, by three main mechanisms [7]: generalized, specialized and lateral transduction (phage mediated) [8]; transformation (transfer of nacked DNA); conjugation (transfer by direct cell-to-cell contact) [9]. The emergence of resistance to different types of drug (MDR) in the same bacterial cell allowed to identify mobile genetic elements, transposons and integrons [10], whose special role is to develope and spread MDR among bacteria of clinical relevance.

It is now acknowledged that most of microorganisms grow in the form of biofilms. More than 80% of all microbial infections are biofilm-related, as shown by the group of bacteria that form biofilms called ESKAPE (*Enterococcus faecalis*, *Staphylococcus aureus*, *Klebsiella pneumoniae*, *Acinetobacter baumannii*, *P. aeruginosa* and *Enterobacter* spp.), responsible for high mortality [11]. Biofilms are made of well-structured aggregations of single or multi-species bacteria, which cooperate with each other and their environment, within polymeric matrices produced by cells themselves and attached to biological surfaces or inerts. They have different morphologies depending on the bacterial strains and growth conditions and are responsible for virulent and chronic infections. The classic developmental model of biofilm formation consists of several distinct phases. In a first stage, single cells associate with a solid surface by adhesion; then, in the second stage, they aggregate in micro colonies, usually composed of many types of micro-communities, surrounded by self-produced extracellular polymeric substance (EPS), such as proteins (<1–2%) including enzymes), DNA (<1%), polysaccharides (1–2%) and RNA (<1%). In addition to these components, water (up to 97%) is the major part of biofilms and is responsible for the flow of nutrients inside the biofilm matrix. In the final stage, a detachment from mature biofilm (as planktonic bacteria) in the environment may occur; sometimes, a mechanical stress may also be involved in this process [12].

Actually, bacterial biofilms exhibit an enhanced resistance to drug therapies and chemical disinfection, and are more resistant to the immune system. This increased resistance is caused by many factors: polymeric matrices protect cells and, as a result, antimicrobial drugs can hardly penetrate the biofilm; concentration gradients of oxygen and nutrients in the biofilm lead to cell differentiation; metabolic cooperation may cause expression of different antibiotic resistance genes that allow bacteria to resist to the presence of high concentrations [13] of antimicrobial.

## 2. Nanomaterials as Antibacterial Agents

The term nanomaterials is used to indicate substances made of objects (nanoparticles NPs) where at least one dimension is less than 100 nm. At this scale, they exhibit unique optical, magnetic and electrical properties (different from the bulk material) that have great impact in many fields. The combination of nanotechnology and biology opens new opportunities in biological and nanomedicine applications and offers a potential solution to biological problems such as infection control and instrument sterilization. They may have a strong catalytic activity, high toxicity and can be exploited for novel applications when properties of atomic or bulk materials are unsuitable.

Many nanomaterials exhibit antimicrobial properties and for this reason they may be used to control microbial populations [14,15,16]. Bacteria are essential to biodiversity on earth, but, as reported in the introduction (Section 1), they are also cause of lethal infections for the human being. They can be exposed to NPs in their natural environment without any adverse effect; for example, the flora of the skin can be exposed to large quantities of NPs incorporated into topical preparations and cosmetics [17,18,19,20,21,22].

**Figure:**
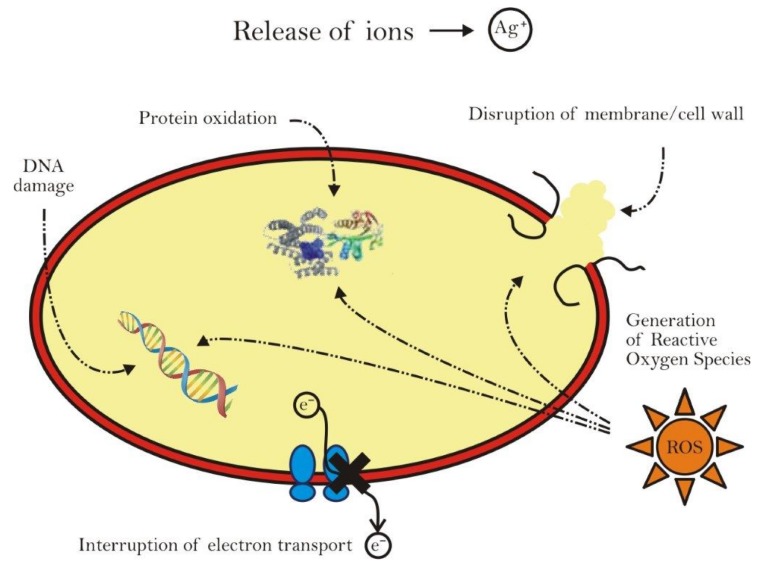
The formation of reactive oxygen species (ROS) leading to cell membrane, protein and DNA damage. NPs intracellular localization has effects on cell reproduction and development, on DNA and it may interfere with the pumping activity of the protein; moreover it causes an increase in cell permeability and an uncontrolled transport through the cytoplasmic membrane and, ultimately, cell death. Ag^+^ leads inhibition of the electron transport.

On the other hand, anti-bacterial NPs have been synthesized from metals, like gold [23], silver [24] copper [25] and iron [26], and from oxides, like cerium oxide [27], zinc oxide [28], titanium dioxide [29] and silicon dioxide [30]. In particular, gold nanoparticles (GNPs) are relatively inert but they become toxic if coated with ligands such as ionic surfactants or antibiotics, which give them antibacterial proprieties [23,31].

As a matter of fact, many studies of bionanotechnology are focused on medical applications, where NPs can be used as anti-bacterials, either independently or in combination with other treatments. A synergistic toxic effect of silver nanoparticles (AgNPs) and antibiotics was observed when tested against *E. coli*. Originally this was reported for amoxicillin [32]; later on, the observation was extended to include enhancement of effects of penicillin, erythromycin, clindamycin and vancomycin [33]; the study reported similar effects against *S. aureus*. Interestingly, both organisms frequently exhibit antibiotic resistance [34]. The effect is not restricted to silver. Zinc oxide NPs increase the efficacy of ciprofloxacin against *E. coli* [28]. Vancomycin, conjugated directly to the surface of GNPs, has more efficient activity compared to vancomycin alone; in this case, the observed synergistic effects occur because of an enhanced reactivity of molecules located at the GNPs surface.

NPs can also have effects in an indirect way, when bacteria must respond to challenging nutrient limitation. Ag and TiO_2_ NPs proved to be toxic to bacteria in NPs coated surgical masks [29]; AgNPs are active in water purification filters [35]; Experiment with *E. coli* show that AgNPs can cause cell lysis and cell shrinking [36], with a significant inhibition of the cell growth. A recent hypothesis on the action of silver NPs is that their degree of toxicity is proportional to the release of Ag^+^ [24], an antimicrobial cation. AgNPs can also produce free radicals [37], with a diminished activity in presence of an anti-oxidant. Redox-active NPs of iron, or composite NPs containig ferrous iron (Fe_2_^+^), inhibit *E. coli* growth on solid medium [26]. Also TiO_2_, SiO_2_ and ZnO NPs exhibit an oxidative, stress-based, toxic effect [30].

## 3. Theoretical Description of Induced Temperature Variations

The study of the interaction of light with nanosized objects represents an important issue not only for photonics and plasmonics but also for physics, chemistry, biology and nanomedicine. In application areas, metallic NPs are of high interest because they possess an intrinsic ability to confine light at the nanoscale through the excitation of Localized Plasmonic Resonance (LPR), a phenomenon related to the formation of plasmons, that are oscillations of the free electrons localized at the metal/dielectric interface. Under a resonant light illumination, metallic nanoparticles (MNPs) convert light into heat, thus, becoming nanosources of heat, a phenomenon that opens up an unpredictable number of applications, ranging from photonics to nanomedicine [38,39]. The effect is due to the absorption associated to LPR formation, which triggers a heat generation process; this involves not only absorption of incident photons, but also heat transfer from MNPs to the surrounding medium. In this framework, Gold nanoparticles (GNPs) have gained an important role because their LPR can be tuned from the visible to the near-infrared (NIR) range by simply changing their size, shape, or the dielectric function of the surrounding medium. Moreover, GNPs are bio-compatible and can be easily functionalized with a large variety of molecules, also exhibiting a weak oxidation. Thanks to their properties, GNPs have been widely exploited in cancer therapy [40,41,42,43,44], ophthalmology and dermatology, nanosurgery [45,46,47,48,49], photothermal imaging [50], plasmon-assisted nanochemistry [51], plasmon-assisted optofluidics [52] and sensing [53,54].

**Figure 2:**
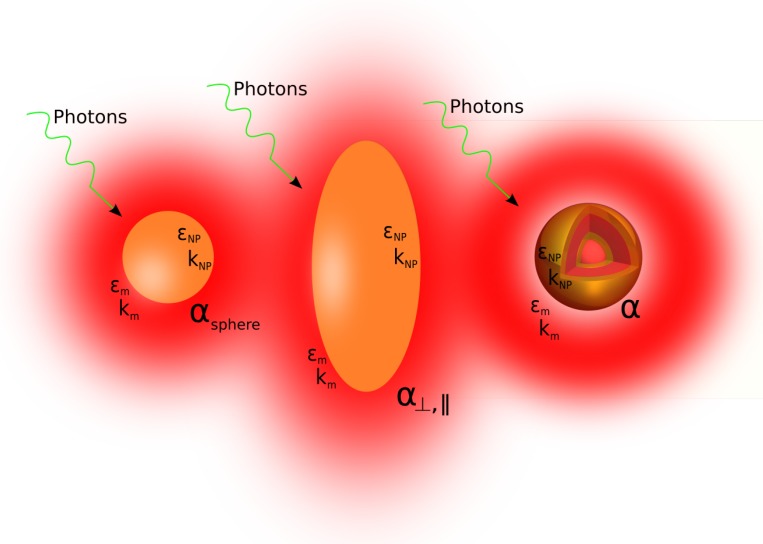
Sphere, ellipse and a complex structure of metal nanoparticles. Temperature variation around the MNP can be described through polarizability α, a parameter that depends on shape, structure and metal that the MNP is made of (dielectric permittivity $\varepsilon_{NP}$ and thermal conductivity $k_{NP}$) as well as on the surrounding medium (dielectric permittivity $\varepsilon_{m}$ and thermal conductivity $k_{m}$).

Under the action of an impinging, resonant, electric field, due to the LPR onset, a single MNP produces heat by Joule effect (see Figure 2); thus, in order to describe its temperature variation, it is necessary to known its electric response function, that is its polarizability α. This appears in the relation between the dipole moment p induced in the MNP and the electric field E of the impinging electromagnetic wave:

(1) p=αE

α depends on shape, structure and nature of the surrounding medium as well as on the metal that the MNP is made of.

The conversion efficiency of electromagnetic energy into heat depends on the MNP dimension and, for small nanoparticles, can reach values as high as 100%. In the absence of phase transitions, if α is known, it is possible to predict the value of temperature around a MNP by solving the heat transfer equation (Equation (2)), which derives from a heat energy balance: the net rate of thermal energy that comes out from the MNP, plus the rate of thermal energy accumulation (internal energy variation in the MNP) must equal the net rate of thermal energy generation:

(2) $$\nabla \cdot \left(-k(r)\nabla T(r,t)\right)+\rho \left(r\right)c\left(r\right)\frac{\partial T(r,t)}{\partial t}=Q\left(r,t\right)$$

where r and *t* are the spatial and time coordinates, $T\left(r,t\right)$ is the local temperature and parameters k(r), ρ(r), c(r) indicate thermal conductivity, mass density and specific heat, respectively. The function Q(r,t) represents the source of energy density, coming from the energy dissipation inside the MNP. At the thermal equilibrium, solution of the Equation (2) is obtained by taking into account that heat production takes place at a constant rate per unit time and per unit volume [55]. Calculations, carried out in spherical coordinates, yield a temperature variation at a distance *r* from the MNP given by:

(3) $$\Delta T=\frac{q}{4\pi K_{m}r}$$

where $K_{m}$ is the thermal conductivity of the surrounding medium and the thermal energy generation *q* is given by

(4) $$q=QV_{NP}=<J\left(r,t\right)\cdot E\left(r,t\right){>}_{t}V_{NP}=\sigma_{abs}I=Kℑm(\alpha )I$$

where $V_{NP}$ is the total volume of the MNP, $\sigma_{abs}$ is the absorption cross section of the MNP, *K* is the wavevector of the impinging light of intensity *I*, $<J\left(r,t\right)\cdot E\left(r,t\right){>}_{t}$ is the energy converted into heat due to joule effect into the metal ($<\ldots {>}_{t}$ indicates the time average and ℑm(α) is the imaginary part of α). Equation (4) tells us that, if laser wavelength and intensity and structure polarizability are known, it is possible to calculate thermal energy generation. In order to obtain the polarizability of small MNPs (smaller than the wavelength), it is necessary to solve the Laplace equation by imposing regularity condition in r=0, reconnection to external field in r→∞ and tangential and normal continuity of the electric field (E) and electric displacement (D) at the interfaces. For spherical and rod-like shapes (see Figure 2), it is possible to obtain an analytical expression for the polarizability α whereas for complex structures like nano-matryoshkas or onion-like structure it is necessary to resort to a numerical calculation. In spherical coordinates, polarizability α [56] is given by

(5) $$\alpha_{sphere}=3\varepsilon_{m}V_{NP}\frac{\varepsilon_{NP}-\varepsilon_{m}}{\varepsilon_{NP}+2\varepsilon_{m}}.$$

where $V_{NP}$ is the total MNP volume, $\varepsilon_{NP}$ is MNP permittivity, $\varepsilon_{m}$ is the host medium permittivity. Equation (5) shows that the resonance of α depends on the dielectric permittivity of the surrounding medium $\varepsilon_{m}$ through the Fröhlich condition involving the real part of $\varepsilon_{NP}$: $ℜ\left[\varepsilon_{NP}\right]=-2\varepsilon_{m}$.

The polarizability of a metal nanorod can be calculated by using a prolate spheroid coordinate in the dipole approximation [57], which yields $\alpha_{⊥}$ and $\alpha_{∥}$:

(6) $$\alpha_{⊥,∥}=3\varepsilon_{m}V_{NP}\frac{\varepsilon_{NP}-\varepsilon_{m}}{3\varepsilon_{m}+3L_{⊥,∥}\left(\varepsilon_{NP}-\varepsilon_{m}\right)}$$

where ⊥ and ∥ indicates if the direction of light polarization is perpendicular or parallel to the nanorod long axis; $L_{⊥,∥}$ are geometrical factors given by:

(7) $$\begin{array}{cc} L_{∥} & =\frac{1-e^{2}}{e^{2}}\left(-1+\frac{1}{2e}\ln\frac{1+e}{1-e}\right)\\ L_{⊥} & =\frac{1-L_{∥}}{2} \end{array}$$

*e* is the eccentricity ($e^{2}=1-1/\rho$ where ρ is the aspect ratio of the spheroid ratio between long axis and short axis).

Calculation of the polarizability of nano-matryoshkas like or onion like nanostructures [58], due to the presence of many interfaces, requires to solve an equation system; if the structure symmetry is known, the best way to calculate the polarizability is to use a numerical approach.

**Figure 3:**
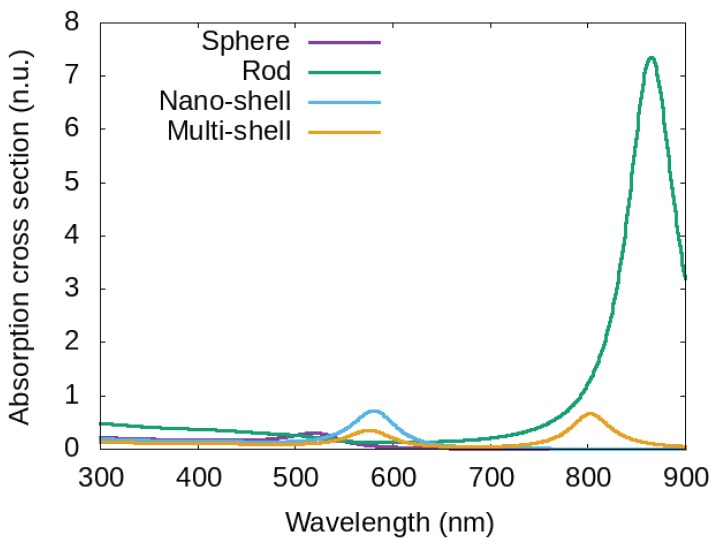
Curves represent the absorption cross section of four MNPs with the same total volume and immersed in water: purple line for a gold nano-sphere with radius 10 nm, green line for a gold nano-rod with long axis 16 nm and short axis ∼7.9 nm, blue line for a nano-shell made of a silica core (radius 7 nm) and a gold shell of 3 nm, Yellow line for a multi-shell structure obtained starting from a dielectric core and alternating gold/dielectric shell with total radius 10 nm.

In order to provide a possibility of comparison, we report in Figure 3 the absorption cross section $\sigma_{abs}$ of four MNPs surrounded by water, with the same total volume, but different shapes and/or structures: a gold nano sphere with radius $R_{np}=10$ nm, a gold nanorod with the same total volume (16 nm and ∼7.9 nm for long and short axis respectively), a nano-shell made of a silica-core (with radius 7 nm and a gold shell of 3 nm), and a multi-shell structure (onion-like) with total radius 10 nm obtained by starting from a dielectric core and alternating gold/dielectric shells.

**Figure 4:**
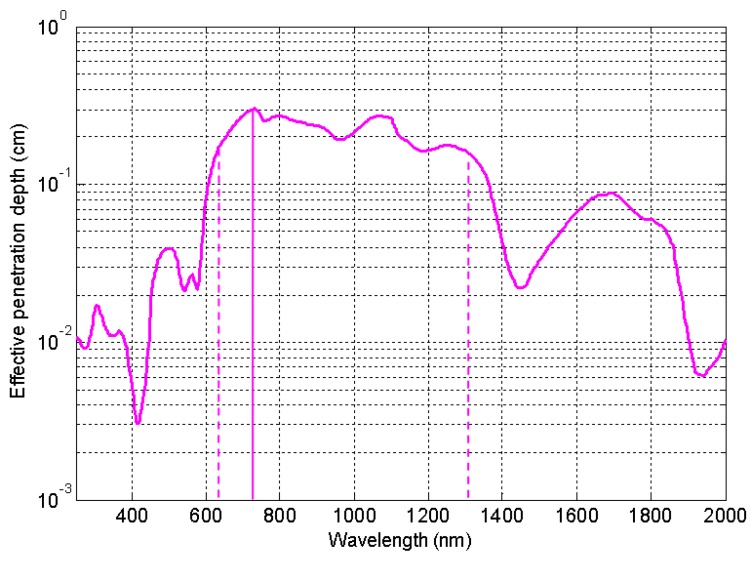
Effective penetration depth in tissue Near-infrared window in biological tissue (626–1316 nm).

Curves of Figure 4 show a plasmonic peak at different wavelength: $\lambda_{peak}=520$ nm for the sphere, $\lambda_{peak}=865$ nm for the rod, $\lambda_{peak}=580$ nm for the nano-shell and two peaks for the multi-shell $\lambda_{peak,1}=576$ nm and $\lambda_{peak,2}=803$ nm. The position of the plasmonic peak of the absorption cross section $\sigma_{abs}$ provides information on the best working wavelength and on the produced heat. Indeed, by starting from the value of $\sigma_{abs}$ at a particular working wavelength, it is possible to calculate the localized temperature around the MNP (see Figure 2 and Equation (4)). By changing size, metal, structure and surrounding medium (dielectric permittivity $\varepsilon_{m}$ and thermal conductivity $k_{m}$) it is possible to change the temperature around MNPs [56,59].

It is important to take into account that the effective temperature depends on the number of MNPs acted on by the electromagnetic wave, because each MNP contributes to the heating process through a heat transfer to the surrounding medium. Therefore the temperature will depend on the arrangement (in a line, a surface or a bulk) and on the MNP density [59].

The heating process so far described can be usefully used to obtain an anti-microbial effect, resulting from a temperature variation in mesophilic bacteria as reported in the following Section 4.

## 4. Anti-Microbial Effect of the Plasmonic Photothermal Heating of Gold Nanoparticles

As already said, anti-bacterial properties of NPs are extensively used in biology. In most cases, the anti-microbial effect of NPs is due to their toxicity, which can be intrinsic (due to the NPs effect directly: Ag or TiO_2_) or due to a coating effect (see Section 2). In addition, a new approach is possible, in which no toxicity is involved. If we consider mesophilic bacteria, that grow at a moderate temperature (typically between 20 °C and 45 °C) it is possible to use the thermo-plasmonic effect of MNPs as an antibacterial mean, without triggering any toxic effect. In particular, the heating mechanism of MNPs, described in Section 3, shows how the used metal, shape, structure and surrounding medium [60,61] play the main role in the heating process.

The photothermal-driven approach has been used in [62] where gold nanorods have been exposed to near-infrared radiation and a significant reduction in bacterial cell viability [62] has been observed. It is shown that it was possible to selectively target and destroy a pathogenic Gram-negative bacterium, *Pseudomonas aeruginosa* by using gold nanorods covalently linked to primary antibodies.

A similar bactericidal and antibiofilm activity of gold nanorods (AuNRs) against oral microorganisms, obtained by using plasmonic photothermal therapy (PPTT), is reported in [63]. The efficacy of PPTT was obtained by reaching a temperature as high as 67 °C.

A rapid photothermal bacterial inactivation technique has been developed by Santos [64], by irradiating near-infrared (NIR) light onto bacterial cells (*Escherichia coli*, *Bacillus subtilis*, *Exiguobacterium* sp. AT1B) deposited on surfaces coated with a dense, randomly distributed array of nanoporous gold disks (NPGDs). With the use of cell viability tests and SEM imaging analysis, a complete inactivation of the pathogenic bacteria is confirmed within ∼25 s of irradiation of the NPGD substrate.

Gold nanocrosses [65], conjugated to secondary and primary antibodies, completely destroy *P. aeruginosa* and its biofilms upon near-infrared electromagnetic irradiation for 5 min with a λ=800 nm laser at low power density (∼3.0 W cm^−2^). No bacterial activity has been detected after 48 h post irradiation, indicating that the heat generated by the irradiated gold nanocrosses attached to bacteria was effective in eliminating and preventing the re-growth of bacteria. Overall, the conjugated gold nanocrosses allowed an effective photothermal ablation of multidrug-resistant bacteria and their biofilms in a targeted, well localized region, with reduced nonspecific damage to healthy tissue.

By using a LED emission [66] ($\lambda_{peak}=850$ nm) to irradiate gold nanorods, a 71% of the early biofilm of bacteria elimination has been shown after 5 min of irradiation, a result that shows the potential of this novel antibiofilm technique. All above studies demonstrate that plasmonic generated heat of MNPs offers a novel way to eliminate bacterial biofilms. As a perspective, in future applications this method could also be used to eliminate bacterial contamination during implant surgery or simply could represent a way to obtain solar photothermal disinfection [67].

The possibility to change the wavelength of the plasmonic peak by changing size, shape, structure or surrounding medium makes the photoheating of MNPs a very usefull tool for antibacterial activities (see Section 3). Using equations reported in Section 3 it is possible to take into account if the bacteria are immersed in a water solution (refractive index 1.33) or in a tissue (refractive indices from 1.35 to 1.55). For a nano-shell in a tissue with refractive index 1.55, calculations show a plasmonic peak shift of about 30 nm with respect to the peak position in water and similarly for the peaks of a multi-shell, while, for a nanorod in a tissue, the calculation provides a plasmonic peak shift of more then 100 nm with respect to the peak position in water. This high sensitivity of rods to the surrounding medium is experimentally reported in [68]. Looking at Figure 4 it appears evident that, if bacteria are in a tissue, it is necessary to choose a structure whose plasmonic peak wavelength stands in the bio-window (from 626 nm to 1316 nm), so that the penetration depth results satisfactory. Then, the value of the peak position allows to calculate the local temperature increase (see Section 3, Equation (4)). It is possible to change the absorption (and then the temperature variation) by changing size of the MNPs or their eccentricity (in nanorods) or the ratio between the shell rays in the multi shell (onion-like NPs). In all these cases, it is possible to manage the temperature increase, locally induced around the MNPs.

The desired temperature to completely destroy bacteria depends on the specific bacteria and on the exposition time; in general, for mesophilic bacteria, it is possible to completely destroy bacteria in the range 50–60 °C. It is worth pointing out that, since the reached photo induced temperature depends on all the parameters previously cited (shape, structure, surrounding, ⋯) and on the NPs density, each specific case requires an “ad hoc” solution.

## 5. Discussion

In all previous works, where heat is used as an antimicrobial agent, there is no detailed analysis of the intrinsic toxicity of MNPs and then the percentage of effects due to heat and toxicity remains unknown. Thus, if we want to exclude effects of toxicity, in addition to a coating material that has no toxic activity, it is necessary to properly choose the metal which MNPs are made of, their dimensions and the plasmonic resonant wavelength, by properly choosing shape and/or structure. The main requirement for biological applications is related to the possibility to work with a plasmonic resonant wavelength falling in the bio window (where the human body is transparent) in order to reach all MNPs injected in the body (see Figure 4);

this imposes to have the plasmonic resonance in the IR region. This is achievable by using gold nanorods [62,63,66], gold nanocrosses [65], gold nanodisk [64] or more complicated structures like onion-like or nanomatryoshka [58] structures: in all of these differently shaped MNPs it is possible to have a plasmonic resonance (and a following heating process) in the IR region. From a theoretical point of view, it is possible to predict temperature variations around one or more MNPs by means of analytical or numerical analysis. The model can be used to calculate the optimal MNPs concentration needed to obtain the desired effect.

## 6. Conclusions

In conclusion, we have analyzed the phenomenon of the antibiotical resistence, arguing that the problem of multidrug resistance is related not only to the indiscriminate widespread use and abuse of antibiotic, but also to the intrinsic capacity of bacteria to survive in a hostile environment, as a consequence of their genetic plasticity. In fact, in addition to chromosomal mutations, bacteria have sophisticated mechanisms to acquire, transfer and spread resistance genes. We present a tracking shot of possible solutions to this problem through a nanotechnology-driven approach, showing that metal NPs can be used to obtain an antibacterial effect, both for their toxic action and for an induced photo-thermal action. We report that the intrinsic toxic action of Ag and TiO_2_ or the induced toxicity of a NPs coating with ligands (such as ionic surfactants or antibiotics) are able to exert an antimicrobial activity. We also show that, for mesophilic bacteria, a new approach is possible, in which no toxicity is involved. By using the thermo-plasmonic effect of metal nanoparticles, it is possible to completely destroy bacteria and their biofilms, preventing also their re-growth. Furthermore, it is possible to selectively target and destroy bacteria covalently linking the MNPs to primary antibodies. The importance of MNP shape, structure, metal which they are made of, as well as the nature of the surrounding medium, is reported and analyzed. A usefully implemented theoretical model enables to design those MNPs that prove more suitable to obtain the desired effect. 

## Abbreviations

The following abbreviations are used in this manuscript:

MDR	multidrug resistence
EEA	European Economic Area
HGT	Horizontal gene transfer
NP	nanoparticle
GNP	gold nanoparticle
MNP	metal nanoparticle
LPR	localized plasmonic resonance
IR	infrared
NIR	near infrared
